# HIV Drug Resistance Profile in Clients Experiencing Treatment Failure After the Transition to a Dolutegravir-Based First-Line Antiretroviral Treatment Regimen in Mozambique

**DOI:** 10.3390/pathogens14010048

**Published:** 2025-01-09

**Authors:** Nalia Ismael, Cidia Hussein, Cacildo Magul, Humberto Inguane, Aleny Couto, Amancio Nhangave, Ana Muteerwa, Mahoudo Bonou, Artur Ramos, Peter Wesley Young, Sonia Chilundo, Rhoderick Machekano, Lauren Greenberg, Juliana da Silva, Nilesh Bhatt

**Affiliations:** 1 Instituto Nacional de Saúde, Estrada Nacional N1, Marracuene 3943, Mozambique; 2 Division of Medical Virology, Faculty of Medicine and Health Sciences, Stellenbosch University, Cape Town 8000, South Africa; 3 Elizabeth Glaser Pediatric AIDS Foundation, Avenida Paulo Samuel KanKhomba, 280 Bairro da Sommerschield, Maputo 20005, Mozambique; 4 National HIV and STI Control Program, Ministry of Health, Salvador Allende 1008, Maputo 1101, Mozambique; 5 Operational Research Unit, Gaza Provincial Health Directorate, Rua do Hospital Provincial, 33 Bairro 13, Cidade de Xai-Xai 1200, Mozambique; 6 Division of Global HIV & TB, U.S. Centers for Diseases Control and Prevention, Avenida Marginal 5467, Maputo 1101, Mozambique; 7 Elizabeth Glaser Pediatric AIDS Foundation, Washington, DC 20005, USA; 8 Division of Global HIV & TB, U.S. Centers for Diseases Control and Prevention, Atlanta, GA 30333, USA

**Keywords:** HIV, drug resistance, dolutegravir, Mozambique

## Abstract

Real-world data on HIV drug resistance (HIVDR) after transitioning to tenofovir disoproxil fumarate/lamivudine/dolutegravir (TLD) are limited. We assessed HIVDR rates and patterns in clients with virological failure (VF) after switching from an NNRTI-based regimen to TLD. A cross-sectional study was conducted in Gaza, Mozambique (August 2021–February 2022), including adults on first-line ART for ≥12 months who transitioned to TLD and had unsuppressed viral load (VL) ≥ 1000 copies/mL six months post-transition. After three adherence counseling sessions, participants with VF underwent genotyping for drug resistance mutations (DRMs) using the Stanford HIVdb Program. Of 717 participants (median age 39.2 years, 70.7% female), 217 (30.2%) had VF, 193 (88.9%) underwent genotyping, with 183 (94.8%) successfully genotyped. Intermediate–high dolutegravir (DTG) resistance was found in 19.6% (36/183). Unsuppressed VL before DTG transition was independently associated with VF (aOR: 2.14). Resistance patterns included 33.3% (12/36; 95% CI: 14.6–46.3) to all three TLD drugs, 55.6% (20/36; 95% CI: 39.3–71.9) to DTG and 3TC, and 11% (4/36; 95% CI: 0.8–21.3) to DTG only. Major drug resistance mutations to DTG included G118R (9.3%), R263K (6.6%), and Q148H/R/K (4.4%). This study highlights the need to consider virologic status before transitioning PLHIV to TLD and suggests that adherence counseling may not prevent resistance in those with unknown or prior VF.

## 1. Introduction

In 2019, the World Health Organization (WHO) updated the guidelines recommending a transition from non-nucleoside reverse transcriptase (NNRTI) to integrase strand transfer inhibitor (INSTI)-based antiretroviral treatment (ART) regimens specifically for low- and middle-income countries [1]. In many countries, particularly in sub-Saharan Africa, dolutegravir (DTG) in combination with two nucleoside reverse transcriptase inhibitors (NRTIs) is now the standard of care for adults and children.

Aligned with WHO recommendations, in 2019 the Mozambique National HIV and STI Control Program introduced DTG-based ART regimens for adults as first-line ART. Adults on first-line ART with tenofovir disoproxil fumarate (TDF)/lamivudine (3TC)/efavirenz (EFV) (TLE) were transitioned to TLD (TDF/3TC/DTG) irrespective of virologic status. The introduction of TLD was followed by improved HIV-1 viral suppression among adults defined as load <1000 copies/mL, from 55% in 2018 during the pre-DTG introduction to 89% after the rapid DTG transition roll-out in 2022 [2]. Despite these impressive gains in suppression rates, there is still limited real-world data from sub-Saharan Africa about the risk of emerging drug resistance to DTG, especially in settings where HIV drug resistance testing is not routinely done. Prior to the introduction of INSTI in Mozambique, a study on treatment-naïve patients found no major drug resistance mutations to INSTI, indicating this class of ARVs could be optimal for people initiating treatment for the first time. Likewise, data from West Africa and South Asia have also reported a low frequency of major drug resistance of INSTI in pre-treatment patients, with rates below 2%. These findings support the introduction of such ARVs in limited-resource countries to improve treatment outcomes [3,4]. However, a study from Gabon has reported a higher frequency of major drug resistance among treatment-naïve patients, estimated at 9%. Most of the major drug resistance mutations were reported for the first generation INSTI, raltegravir (RAL) and elvitegravir (EVG) [5], where information on the impact of such mutations on second-generation INSTIs such as DTG, cabotegravir (CAB), and bictegravir (BIC) remains scarce. Other studies published by Breneer et al. and Loosli et al. also showed that prior NRTI mutations can increase the risk of acquired DTG drug resistance [6,7]. Moreover, naturally occurring polymorphisms that vary within HIV subtypes have been associated with different mutational pathways affecting the levels of INSTI resistance [8,9]. All these studies only emphasize the need for more real-world data on resistance profiles following the transition to a DTG-based regimen, particularly in sub-Saharan Africa where non-B subtypes predominate.

Given the widespread use of DTG-based regimens and the limited routine use of HIV drug resistance testing, we assessed the emergence of DTG resistance among highly treatment-experienced patients experiencing virologic failure after transition to TLD in a programmatic setting in Mozambique.

## 2. Materials and Methods

### 2.1. Study Design and Population

A cross-sectional study was conducted between July 2021 and February 2022 in seven high-volume (defined as >2000 clients on ART) public health facilities in Gaza province, southern Mozambique. We enrolled adult clients (age ≥ 18 years) with confirmed HIV-1 infection, history of receiving another first-line ART regimen that was non-DTG-based for at least 12 months before switching to TLD, and who returned to the facility for a viral load (VL) measure after (a) having had an initial VL ≥ 1000 copies/mL at least six months after transitioning to TLD, (b) completing at least three enhanced adherence counseling (EAC) sessions, and (c) allowing at least three months and no more than nine months between VL blood draws. Virological failure (VF) was defined as two consecutive VL measurements with VL ≥ 1000 copies/mL at least three months apart, after completing at least three EAC sessions (see Supplementary Figure S1). Six months after transitioning to the DTG-based ART regimen, HIV RNA testing was performed in accordance with the Mozambique Ministry of Health guidelines.

As part of routine service, health facility staff reached out by phone to clients with unsuppressed VL who were due for a repeat VL test at least three months after an initial unsuppressed VL. For clients who returned for a follow-up clinical appointment, eligibility screening was performed, and written consent was obtained by the study team. At enrolment, client-level information was abstracted from electronic files and/or medical charts at each site. Unclear or missing data in the medical charts were reviewed or confirmed with the participants at the time of enrolment. Socio-demographic data (age, sex, employment status) and clinical laboratory data (ART regimen at initiation and currently; dates of ART initiation and switch; VL results before and after the switch to TLD; enrolment in differentiated service delivery models; and prior or current active TB infection) were collected through a participant interview and/or by reviewing medical charts. In addition, socio-behavioral aspects affecting ART adherence were collected for all enrolled participants through a review of participants’ medical charts.

### 2.2. HIV-1 Viral Load Testing

A total of five milliliters (mL) of whole blood were drawn in ethylene diamine tetra-acetic acid (EDTA) tubes from each participant and sent to two provincial molecular biology laboratories located in Gaza province for HIV-1 VL testing. Buffy coat and plasma were separated from remnant blood at the health facilities and/or VL testing laboratories within six hours after sample collection. Plasma samples for enrolled participants were registered on the Laboratory Information System (LIS) (Laboratory System Technologies (Pty) Ltd., Johannesburg, South Africa), and in a separate database for the study. The HIV-1 VL was quantified using Abbott RealTime HIV-1 assay [10] on the Abbott m2000 sp/rt System and COBAS AmpliPrep/COBAS TaqMan HIV-1 Test, v2.0 on the AmpliPrep/Cobas TaqMan (CAP/CTM) (Roche Molecular Diagnostics, Branchburg, NJ, USA, product codes: 05212294190) according to the manufacturer’s instructions [11]. The limit of detection of HIV-1 VL for Abbott was 40 copies/mL and 20 copies/mL for the CAP/CTM. After HIV-1 VL testing, remaining plasma from samples with a VL ≥ 1000 copies/mL was aliquoted in a separated cryovial tube and frozen immediately at −70 °C to −80 °C until their shipment to the Instituto Nacional de Saúde (INS) laboratories in Maputo for HIVDR testing.

### 2.3. HIVDR Sequencing and Analysis

HIVDR genotyping was conducted at the INS reference laboratory in Maputo, Mozambique. HIV RNA was extracted from 140 μL of plasma using the QIAamp Viral RNA Mini Kit (Qiagen, Hilden, Germany) according to the manufacturer’s instructions [12]. Extracted RNA was immediately genotyped. The HIV-1 Genotyping Kit with Integrase (catalogue number A55120, Thermo Fisher Scientific, Warrington, UK) was used to genotype the protease (PR), reverse transcriptase (RT), and integrase (IN) regions of the HIV-1 pol gene [13]. The genotyping result encompasses codons 6–99 of the PR region, codons 1–251 of the RT region, and codons 1–288 of the IN region of the gene. Sequencing was performed using Applied Biosystem (ABI) 3500 genetic analyser (Applied Biosystems, Foster City, CA, USA) following the manufacturer’s instructions. Sequences were edited using Recall software version 2 [14].

Final sequences were stored in the study database and submitted to the Stanford HIVdb (https://hivdb.stanford.edu/hivdb/by-sequences/ accessed on the 22 June 2023) version 8.9.1 to determine HIVDR mutations and drug susceptibility profiles. HIV-1 subtypes were determined by the REGA HIV-1 Subtyping Tool version 3 [15].

### 2.4. Statistical Analysis

We summarized participants’ demographic characteristics using descriptive statistics. We determined the proportion of clients with (<1000 copies/mL) and without viral suppression (≥1000 copies/mL) on the TLD ARV regimen. Odds ratios and associated 95% confidence intervals were used to summarize the strength and direction of association with factors associated with VF and with presence of INSTI drug resistance mutations after DTG transition. Individual drug susceptibility was summarized using proportion by ARV group class. The statistical analysis was conducted using Stata version 17.0.

## 3. Results

### 3.1. Study Design and Population

**Figure 1:**
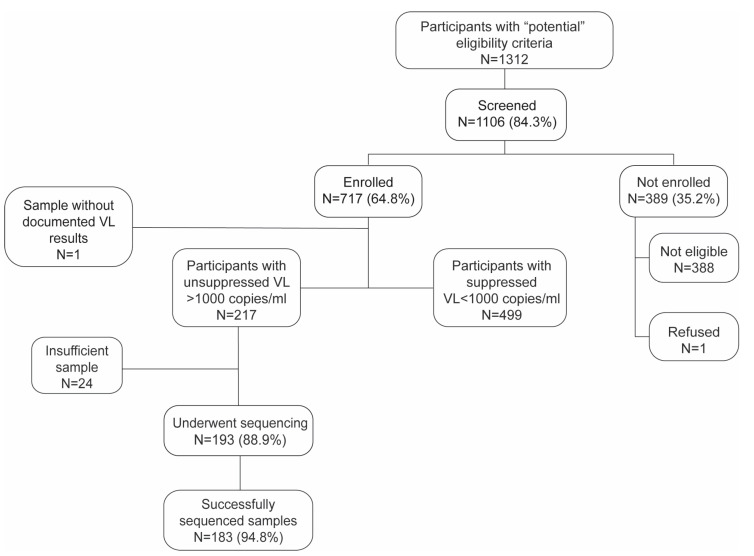
Flow chart of study participants from eligibility assessment to sequence analysis in seven health facilities in Gaza, Southern Mozambique, 2021–2022.

Of the 1312 eligible participants, 1106 (84.3%) were screened and 717 (64.8%) were enrolled in the study. Out of the enrolled participants, 499 (69.6%) achieved subsequent viral load (VL) suppression after three EAC sessions, while the remaining 216 (30.3%) did not (Figure 1). One participant was excluded from the final analysis due to missing documentation of their second viral load measurement. The median age was 39.2 years (IQR: 32.4–46.6), and 506 (70.7%) participants were female; more detailed socio-demographic characteristics are described in Table 1. Among the enrolled participants, 341 (47.5%) had pre-DTG suppressed VL, 162 (22.5%) had no VL test results before the transition, and the remaining 214 (29.8%) had unsuppressed VL pre-DTG. For most participants (78.5%), the initial treatment regimen was TLE, the median time on ART at the time of DTG transition was 5.2 years (IQR: 2.8–7.9), and the time on DTG was 19.7 months (IQR: 16.4–22.8). The median (IQR) plasma HIV RNA level among the 214 clients with recorded VL test results, who were viremic at the time of the switch, was 17,018 copies/mL (IQR: 4636–62,109). In addition, the median time between DTG initiation and first VL (unsuppressed) after the switch was 15.9 months (IQR: 12.3–18.8).

### 3.2. Virologic Failure After DTG Transition

**Figure 2:**
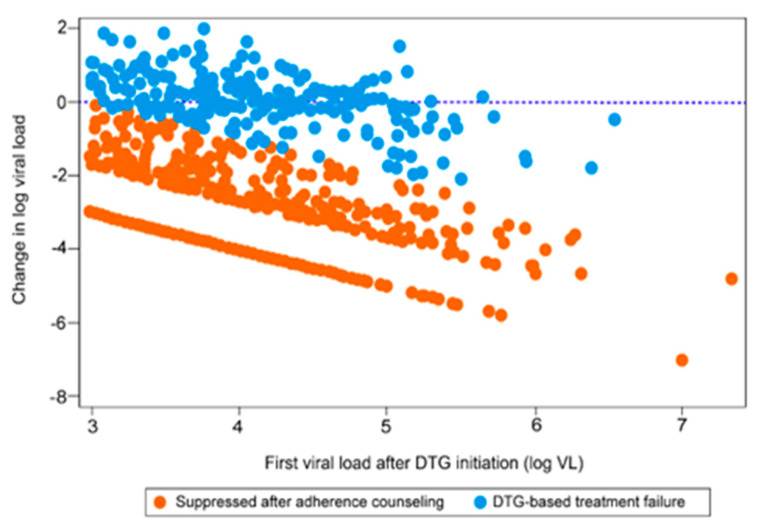
Changes in log viral load between first and second viral load after DTG transition, separated by enhanced adherence counseling.

Figure 2 shows the change in log VL between the first VL after TLD initiation and the subsequent VL separated by three EAC sessions. In general, the mean decrease for all participants was 2.27 log. For the participants that failed TLD (n = 217), the average mean increase was 0.05 log (SD +/− 0.75), and for the participants with suppressed VL, the average decrease was 2.98 log (SD + 1.41) (see Supplementary Table S1). Compared to PLHIV with suppressed VL before DTG-based treatment transitions, those with unsuppressed VL had higher odds of virologic failure (aOR: 2.14, 95% CI: 1.42–3.20), whereas those aged 60 years and above had 70% lower odds of VF when compared to those aged 18–24 years (aOR: 0.30, 95% CI: 0.09, 0.99), indicating a protective effect (Table 2). The prevalence of INSTI resistance mutations after DTG transition was substantially lower in those for whom the initial ART regimen was TDF + 3TC + EFV (OR: 0.16, 95% CI: 0.05, 0.48) (Table 3).

### 3.3. HIV Drug Resistance Mutation and Susceptibility Profile

Of the participants that experienced VF, intermediate- to high-level resistance to DTG was observed in 36 (19.6%; 95% CI: 13.9–25.4), and no low-level DT resistance was observed. Intermediate and high levels of resistance were observed in 11 (6%) and 25 (13.7%) of the study participants, respectively. Major integrase resistance mutations G118R (17, 9.3%), R263K (12, 6.6%), and Q148H/R/K (8, 4.4%) were detected. Other INSTI mutations such as E138K/A/T (22, 12.0%), T66A/I/K (13, 7.1%), G140S/A/C (5, 2.7%), N155H (4, 2.2%), and E92Q (1, 0.5%) were also detected. Resistance to DTG was more common in individuals who had no VL results available before the transition [37.5% (95% CI: 24.8–57.9)] when compared with those who had unsuppressed [17.8% (95% CI: 9.8–28.5)] or suppressed VL before transitioning to DTG-based ART regimen, [11.4% (95% CI: 5.1–21.3)] (see Supplementary Figure S2). Among the participants with DTG resistance, 33.3% (12/36) (95% CI: 14.6–46.3) showed resistance to all three ARVs in the TLD regimen, 55.6% (20/36) (95% CI: 39.3–71.9) to DTG and 3TC, and 11% (95% CI: 0.8–21.3) only to DTG. None of the participants showed combined resistance to DTG and tenofovir alone.

**Figure 3:**
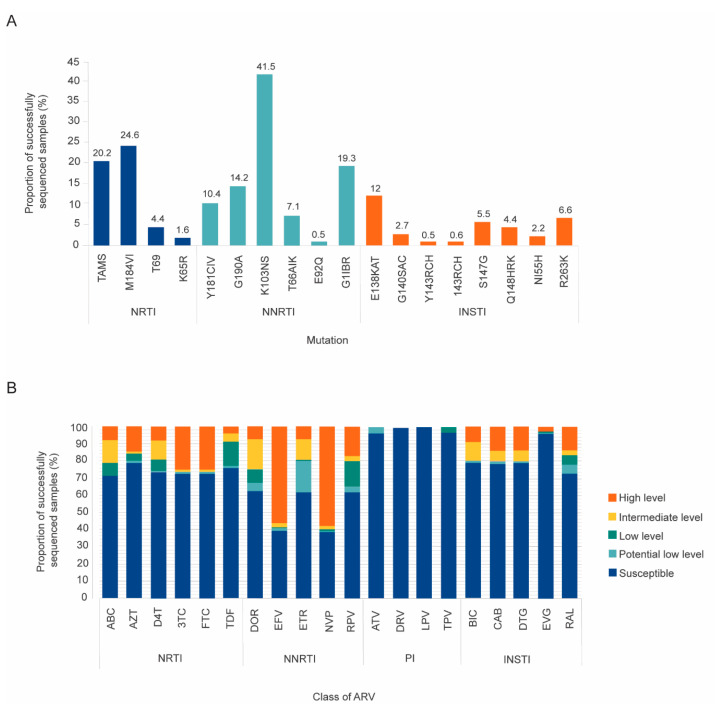
Panel (**A**) Drug resistance mutations to the different classes of ARVs found among the participants experiencing virological failure that were successfully sequenced (n = 183). Panel (**B**) Level of resistance to the different classes of ARVs among the participants experiencing virological failure that were successfully sequenced (n = 183). Mutation profiles and susceptibility predictions were determined using Stanford HIV Drug Resistance Database Version 8.8.0. Proportion of sequences with DRMs to the different classes of ARVs are divided into nucleoside reverse transcriptase inhibitors (NRTIs), non-nucleoside reverse transcriptase inhibitors (NNRTIs), and integrase strand transfer inhibitors (INSTIs) (Panel (**A**)). Individual predicted susceptibility for each antiretroviral (ARV) was categorized according to high-level, intermediate-level, and low-level resistance, and susceptibility (Panel (**B**)). Abbreviations: ARV, antiretroviral therapy; NRTI, nucleoside reverse transcriptase inhibitor; NNRTI, non-nucleoside reverse transcriptase inhibitor; INSTI, integrase strand transfer inhibitor; ABC, abacavir; AZT, azidothymidine (zidovudine); D4T, stavudine; FTC, emtricitabine; 3TC, lamivudine; TDF, tenofovir; DOR, doravirine; EFV, efavirenz; ETR, etravirine; NVP, nevirapine; RPV, rilpivirine; ATV, atazanavir; DRV, darunavir; LPV, lopinavir; TPV, tipranavir; BIC, bictegravir; CAB, cabotegravir; DTG, dolutegravir; EVG, elvitegravir; RAL, raltegravir.

We also detected resistance mutations for NNRTIs, NRTIs, and PIs in 57.9% (n = 106), 30.1% (n = 55), and 3.8% (n = 7) of the participants, respectively. Further description of predicted drug resistance susceptibility can be found in Figure 3. Of note, the common NRTI mutations M184V and K65R were detected, and high-level resistance to 3TC and tenofovir (TDF) was observed in 3.3% (n = 6) and 24.6% (n = 45), respectively. The distribution of all mutations is presented in Figure 3.

With regards to the subtypes C, A, D, and G estimated at 96.7% (n = 177), 1.6 (n = 3), 1.1 (n = 2), and 1% (n = 1) were observed in our participants.

## 4. Discussion

Our study focused exclusively on a treatment-experienced population with confirmed VF, which may explain why our estimates for dolutegravir resistance are higher than those seen in other studies that featured primarily treatment-naïve populations [16,17,18]. The observed levels of resistance to DTG in this sub-population suggest that management strategies may need to differ from those applied in populations receiving TLD as an initial regimen, potentially necessitating a regimen switch.

Several factors may contribute to the high level of DTG resistance in the people living with HIV that transitioned from the previous regimen to TLD. Firstly, the study by Siedner et al. [19] conducted in South Africa suggests that the risk of DTG resistance is higher in those with previously reported NNRTI failure, which might explain the high level of NNRTI resistance observed in almost half (50%) of the participants with VF. Secondly, our results show that unsuppressed VL prior to TLD switch was strongly associated with VF but not DTG resistance, suggesting that virologic status prior to the transition to TLD seems to confer an additional risk for poor treatment outcomes. Lastly, only three (2.3%) individuals showed dual resistance to 3TC and TDF, with the risk of developing functional DTG monotherapy [6]. This is similar to a study conducted in Mozambique that also reported increased acquired resistance levels to the NRTI profile before TLD was implemented [20]. Still, there are contradictory data from different studies regarding the efficacy of DTG-based regimens in patients who transitioned from a non-DTG-based regimen to TLD. In a study from Lesotho, DTG resistance emergence was estimated at 8% in patients who transitioned from an NNRTI-based regimen [21], whereas from Uganda in the Prospective Observational Cohort Study (DISCO), no DTG resistance at 48 weeks after the transition was observed [22].

Moreover, evidence on how the efficacy of a DTG-based regimen is affected by pre-existing NRTI resistance [23] in our study and in patients experiencing VF is also still not well understood. Nevertheless, the presence of NRTI resistance was not associated with DTG resistance. Considering these factors, and in line with the recommendations by Murphy et al. [24], who discuss DTG resistance in the context of African programmatic settings, there is a need for more prospective research to better understand the impact of pre-existing NRTI resistance mutations and other risk factors associated with the development of DTG resistance. Furthermore, such studies must focus on determining the optimal patient management care strategies that can include changes in regimens with anchored PI-boosted regimens for affected patients.

The proportion of individuals that achieved VL suppression after intensive counseling was 69.5% (n = 499), showing the positive impact that adherence interventions can have on treatment outcomes. Nonetheless, 80.4% of our participants with persistent VF had no mutations associated with resistance to DTG, TDF, and 3TC, suggesting adherence, rather than drug resistance, is the primary issue, which could potentially be due to inadequate access to healthcare facilities, stigma, and discrimination common in resource-limited settings [25]. These findings highlight adherence as a major limitation on ART efficacy in the African context, and as suggested by the previous studies, long-acting formulations may be an option to overcome this issue [26,27].

Further, we also observed a very low level of resistance to PIs given the high genetic barrier of this class of ARVs [28], which are only available in second-line regimens in Mozambique and other resource-limited settings. Similar to some countries in the same region, E138K/A/T, G118R, R263K, and Q148H/R/K were the most frequent INSTI DRMs [29]. Notably, the major mutation G118R previously described as a DTG resistance pathway in subtype C [30], also detected in Botswana [31], was also reported at a high rate (10.2%) here. The secondary mutation E138K/A/T in combination with G118R was observed in three participants, previously identified in subtype C [32] and not subtype B in a culture drug selection study demonstrating increased resistance to DTG. Similarly, mutations R263K and Q148H, known to be frequent in subtype B [33] after exposure to raltegravir (RAL), have also been reported in East Africa [29,33], where subtype D/A is common, and were also observed in subtype C infections in our study. Our data highlight the critical need to closely monitor INSTI DRMs, particularly for clients on DTG treatment, to ensure the ongoing success of the transition to DTG-based first-line regimens in sub-Saharan Africa.

Our study has some limitations. Firstly, the inclusion criteria were limited to treatment-experienced populations and should not be extrapolated to populations of all patients experiencing VF while on DTG. Secondly, the Sanger-based sequencing technology can miss 30% or more DRMs, which could potentially further underestimate the true DRM rate among this study population [34]. Thirdly, we only sequenced the pol region of HIV; evidence of mutations outside the integrase that can be associated with DTG resistance has been reported [35,36]. Fourthly, we lacked data on the number of participants with repeat unsuppressed viral loads who achieved suppression, and long-term outcomes such as mortality were not evaluated due to study design constraints. Fifthly, the lack of pre-existing resistance data has limited our understanding of the real impact of major NRTI mutations on subsequent VF after switching to TLD. Likewise, we were unable to determine DRMs in participants with low-level viremia; as such, the emergence of archived major mutations may have been underestimated. Lastly, our study was only conducted in one province of Mozambique, Gaza, which has the highest prevalence of HIV in Mozambique (20.5%) [37] as well as dynamic circular migration patterns; thus, the results cannot be generalized for the whole country, emphasizing the need for similar studies in other regions of Mozambique, including other population groups such as children and pregnant women, to provide a more comprehensive understanding of DTG resistance patterns.

## 5. Conclusions

Our study highlights that almost 20% of sequenced specimens demonstrated intermediate- to high-level DTG resistance among patients with unsuppressed VL on DTG-based ART after switching from a non-DTG-based first-line ART regimen. The findings of this study underscore that most patients with a high viral load should continue to receive a DTG-based regimen but may need strengthened psychosocial support to improve re-suppression rates. Among treatment-experienced patients, better decision tools may help identify PLHIV that harbors resistance and that would benefit from individual drug resistance testing and, eventually, from a drug switch; although history of viral non-suppression was not independently associated with drug resistance, the higher rates observed suggest this may be a criterion to consider for such a decision. As the level of acquired drug resistance for DTG increases as the uptake among PLHIV increases, transmitted DTG resistance in treatment-naïve patients may rise in the long term.

## Figures and Tables

**Table 1:** Clinical and demographic characteristics among participants by virological status during subsequent viral load testing after transition to dolutegravir (DTG), Gaza province, Mozambique, 2021–2022.

Characteristics	Virologic Status at Subsequent VL After DTG					
	<1000 Copies/mL n (%)		≥1000 Copies/mL n (%)		Total N (%)	
**Virologic status at subsequent VL after DTG, copies/mL**						
**<1000**	499	100.0	0	0.0	499	69.7
**≥1000**	0	0.0	217	100.0	217	30.3
**Sex**						
**Female**	348	69.7	158	72.8	506	70.7
**Male**	151	30.3	59	27.2	210	29.3
**Age group, years**						
**18–24**	45	9.0	21	9.7	66	9.2
**25–39**	202	40.5	124	57.1	326	45.5
**40–59**	204	40.9	67	30.9	271	37.8
**60+**	48	9.6	5	2.3	53	7.4
**Median age, years**	41.6	(12.4)	37.3	(10.3)	40.3	(11.9)
**Current employment status**						
**Unemployed**	313	63.9	135	64.6	448	64.1
**Employed**	177	36.1	74	35.4	251	35.9
**Past history of tuberculosis**						
**No**	462	92.6	192	88.9	654	91.5
**Yes**	37	7.4	24	11.1	61	8.5
**Tuberculosis currently active**						
**No**	492	98.6	213	98.6	705	98.6
**Yes**	7	1.4	3	1.4	10	1.4
**Currently in differentiated care models**						
**No**	96	19.2	53	24.4	149	20.8
**Yes**	403	80.8	164	75.6	567	79.2
**Virologic status before switching to DTG, copies/mL**						
**<1000**	257	66.9	83	48.8	340	61.4
**≥1000**	127	33.1	87	51.2	214	38.6
**Initial ART regimen**						
**AZT + 3TC + EFV**	3	0.6	0	0.0	3	0.4
**AZT + 3TC + NVP**	96	19.2	33	15.2	129	18.0
**D4T + 3TC + EFV**	5	1.0	2	0.9	7	1.0
**D4T + 3TC + NVP**	13	2.6	0	0.0	13	1.8
**TDF + 3TC + EFV**	382	76.6	182	83.9	564	78.8

Abbreviations: DTG, dolutegravir; AZT, zidovudine; 3TC, lamivudine; EFV, efavirenz; NVP, nevirapine; D4T, stavudine; TDF, tenofovir; n, number.

**Table 2:** Demographic and clinical characteristics associated with virologic failure after DTG transition, Gaza province, Mozambique, 2021–2022 Abbreviations: log = logarithm; DTG = dolutegravir; VL = viral load.

Characteristics	aOR	95% CI	*p*-Value
**Sex**			
Female	1.00		
Male	0.96	0.59, 1.56	0.870
**Age group, years**			
18–24	1.00		
25–39	1.29	0.67, 2.48	0.443
40–59	0.67	0.34, 1.32	0.245
60+	0.30	0.09, 1.00	0.049
**Current employment status**			
Unemployed	1.00		
Employed	1.25	0.79, 1.97	0.342
**History of tuberculosis**			
No	1.00		
Yes	1.42	0.72, 2.80	0.315
**Tuberculosis currently active**			
No	1.00		
Yes	0.78	0.18, 3.32	0.734
**Currently in differentiated care models**			
No	1.00		
Yes	0.68	0.42, 1.09	0.112
**Virologic status before switching to DTG, copies/mL**			
<1000	1.00		
≥1000	2.14	1.42, 3.20	0.000
Initial ART regimen			
AZT + 3TC + EFV	1.00		
AZT + 3TC + NVP	0.74	0.43, 1.28	0.277
D4T + 3TC + EFV	1.69	0.29, 9.90	0.560
D4T + 3TC + NVP	1.00		
TDF + 3TC + EFV	1.00		

Abbreviations: aOR, adjusted odds ratio; CI, confidence interval; DTG, dolutegravir; AZT, zidovudine; 3TC, lamivudine; EFV, efavirenz; NVP, nevirapine; D4T, stavudine; TDF, tenofovir.

**Table 3:** Factors associated with integrase inhibitor (IN) drug resistance mutations after DTG transition, Gaza province, Mozambique, 2021–2022.

Characteristics	OR	95% CI	*p*-Value
**Sex**			
**Female**	1.00		
**Male**	1.31	0.40, 4.30	0.655
**Age group, years**			
**18–24**	1.00		
**25–39**	0.52	0.12, 2.22	0.378
**40–59**	0.70	0.14, 3.59	0.673
**60+**	2.01	0.08, 52.60	0.676
**Current employment status**			
**Unemployed**	1.00		
**Employed**	0.54	0.17, 1.75	0.302
**Past history of tuberculosis**			
**No**	1.00		
**Yes**	0.48	0.10, 2.40	0.373
**Currently in differentiated care models**			
**No**	1.00		
**Yes**	0.63	0.23, 1.74	0.373
**Virologic status before switching to DTG, copies/mL**			
**<1000**	1.00		
**≥1000**	1.70	0.68, 4.26	0.260
**Initial ART regimen**			
**AZT + 3TC + NVP**	1.00		
**D4T + 3TC + EFV**	1.00		
**TDF + 3TC + EFV**	0.16	0.05, 0.48	0.001

Abbreviations: OR, odds ratio; CI, confidence interval; AZT, azidothymidine (zidovudine); 3TC, lamivudine; TDF, tenofovir; EFV, efavirenz; NVP, nevirapine; D4T, stavudine.

## Data Availability

All pol (covering position 2267–3302) sequences are available on GenBank with the accession number: PP511974–PP512311.

## Supplementary Materials

The following supporting information can be downloaded at: https://www.mdpi.com/article/10.3390/pathogens14010048/s1, Figure S1: Graphical representation of participant selection, sample collection, and laboratory testing; Figure S2: Dolutegravir (DTG) resistance when stratifying by pre-DTG viral load (VL) results divided into unsuppressed, suppressed, and no VL result pre-DTG; Table S1: Log HIV-1 viral load (VL) change after tenofovir fumarate/lamivudine/dolutegravir (TLD) initiation and subsequent VL separated by three enhanced adherence counseling sessions.
