# Supplementary material for: HIV Drug Resistance Profile in Clients Experiencing Treatment Failure After the Transition to a Dolutegravir-Based First-Line Antiretroviral Treatment Regimen in Mozambique
Source: Pathogens. 2025 Jan 9;14(1):48. doi: 10.3390/pathogens14010048 (PMC11769524; doi:10.3390/pathogens14010048)
Supplement: Supplementary file 1 [file pathogens-14-00048-s001.zip › pathogens-3354335-supplementary.pdf]

HIV Drug Resistance Profile in Clients Experiencing Treatment Failure After the Transition to a Dolutegravir-Based First-Line Antiretroviral Treatment Regimen in Mozambique

Supplementary figures and table

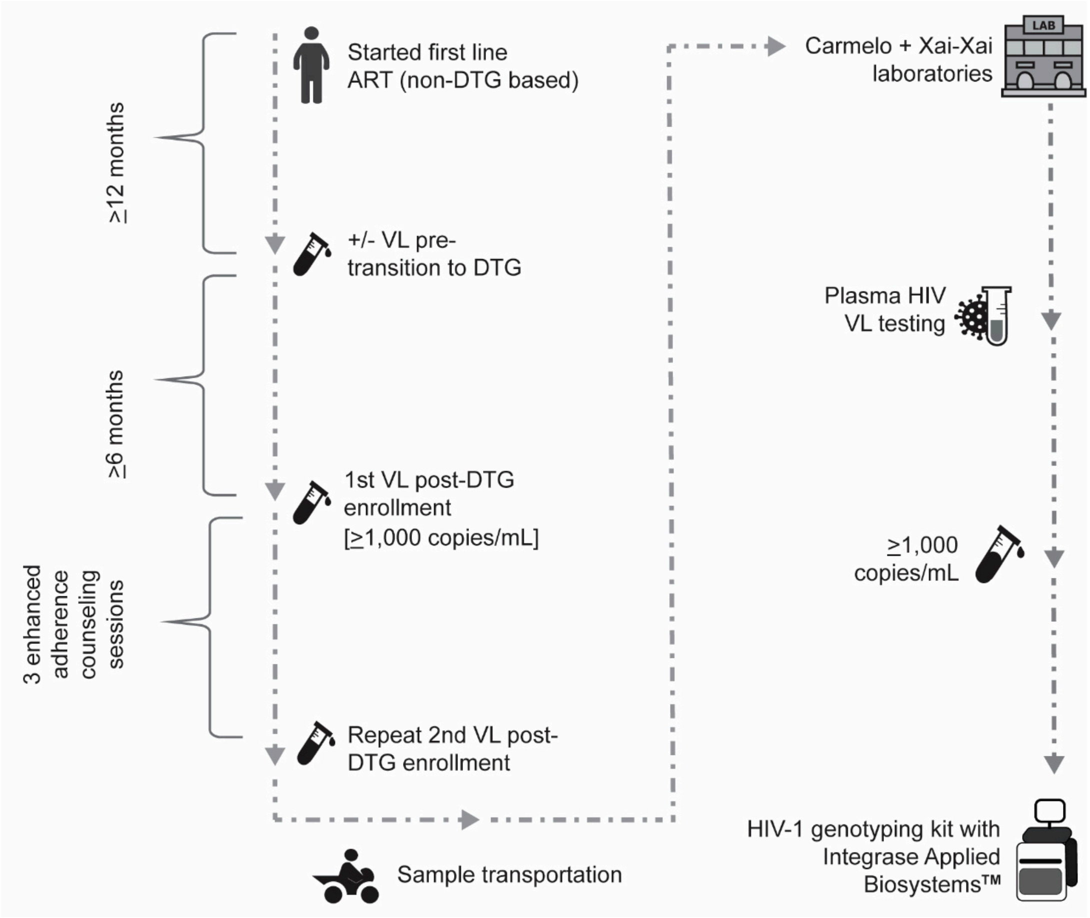

Figure S1: Graphical representation of participant selection, sample collection, and laboratory testing.

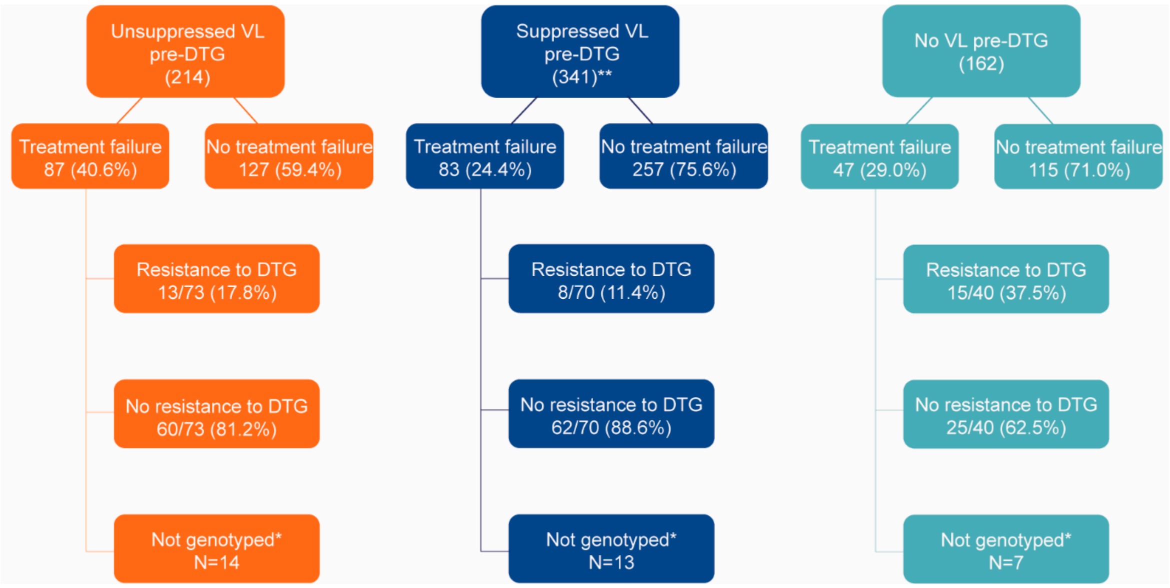

Figure S2: Dolutegravir (DTG) resistance when stratifying by pre-DTG viral load (VL) results divided into unsuppressed, suppressed, and no VL result pre-DTG.

Notes: **No second VL for one sample, *Insufficient sample/failed sequencing.

Table S1: Log HIV-1 viral load (VL) change after tenofovir fumarate/lamivudine/dolutegravir (TLD) initiation and subsequent VL separated by three enhanced adherence counselling sessions.

HIV-1 Viral Load	Mean (SD)	Median (Min, Max)
< 1000 copies/mL	−2.98 (1.41)	−3.14 (−7.0, −0.10)
≥ 1000 copies/mL	0.05 (0.75)	0.06 (−2.10, −1.99)
Total	−2.07 (1.74)	−2.27 (−7.0, −1.99)

Notes: SD=standard deviation; min=minimum value; max= maximum value.
